# Detection of time-, frequency- and direction-resolved communication within brain networks

**DOI:** 10.1038/s41598-018-19707-1

**Published:** 2018-01-29

**Authors:** Barry Crouch, Linda Sommerlade, Peter Veselcic, Gernot Riedel, Björn Schelter, Bettina Platt

**Affiliations:** 1 Institute of Medical Sciences, School of Medicine, Medical Sciences & Nutrition, University of Aberdeen, Foresterhill, Aberdeen, AB25 2ZD United Kingdom; 2 Institute for Complex Systems and Mathematical Biology, University of Aberdeen, King’s College, Old Aberdeen, AB24 3UE United Kingdom; 3 Institute for Pure and Applied Mathematics, University of Aberdeen, King’s College, Old Aberdeen, AB24 3UE United Kingdom; 4 TauRx Therapeutics Ltd, King Street, Aberdeen, United Kingdom; 5 Present Address: AbbVie Deutschland GmbH & Co. KG; Knollstr, 67061 Ludwigshafen, Germany

## Abstract

Electroencephalography (EEG) records fast-changing neuronal signalling and communication and thus can offer a deep understanding of cognitive processes. However, traditional data analyses which employ the Fast-Fourier Transform (FFT) have been of limited use as they do not allow time- and frequency-resolved tracking of brain activity and detection of directional connectivity. Here, we applied advanced qEEG tools using autoregressive (AR) modelling, alongside traditional approaches, to murine data sets from common research scenarios: (a) the effect of age on resting EEG; (b) drug actions on non-rapid eye movement (NREM) sleep EEG (pharmaco-EEG); and (c) dynamic EEG profiles during correct vs incorrect spontaneous alternation responses in the Y-maze. AR analyses of short data strips reliably detected age- and drug-induced spectral EEG changes, while renormalized partial directed coherence (rPDC) reported direction- and time-resolved connectivity dynamics in mice. Our approach allows for the first time inference of behaviour- and stage-dependent data in a time- and frequency-resolved manner, and offers insights into brain networks that underlie working memory processing beyond what can be achieved with traditional methods.

## Introduction

Tracking transient patterns of connectivity in defined behavioural or disease states is one of the most pressing challenges in neuroscience. Functional magnetic resonance imaging (fMRI) offers high spatial resolution, but the slowness of blood oxygen level changes^1^ is at odds with the temporal profile of neuronal signalling. Dynamic neuronal events can be monitored via electroencephalography (EEG) and electrocorticography (ECoG)^2^, yet, harnessing this advantage poses a number of technical challenges as the EEG is fundamentally noisy and derived from non-stationary processes.

Functional connectivity analysis of EEGs is usually based on correlations (coherence) between signals. Until recently, however, the required frequency domain analyses have not been applicable to millisecond time scales^3^. Nonetheless, disruptions of inter-regional EEG connectivity have been reliably observed in numerous neurological disorders including schizophrenia^4^, autism^5^ and Alzheimer’s disease (AD)^6^. However, more comprehensive connectivity assessments are now required which also consider causality and directionality of information transfer, sometimes referred to as ‘effective connectivity’ as opposed to ‘functional’ (correlational) connectivity^7^. For the purpose of this paper, it should be noted that the term “causality” is used based on the notion of predictability in a multivariate framework as defined by Granger^8^.

### Spectral analysis

Regional, spectral analysis of time series is commonly based upon Fourier transformation, typically employing the Fast Fourier transform (FFT). To achieve consistent spectral estimates, windowing of the time series or smoothing of the FFT is required. As frequency resolution is 1/window length, short windows improve temporal resolution but reduce frequency resolution. When choosing the smoothing approach similar restrictions apply. Therefore, the FFT requires a trade-off between frequency and temporal resolution.

An alternative is offered via autoregressive (AR) modelling. Briefly, the AR model assumes that the value of the time series at any given time point is a function of the time series of the previous *n* points and an additive noise contribution. For a linear AR model, the spectrum can be derived from the estimated model parameters. By embedding the AR model into a state space model and augmenting it by adding an equation for the changes in parameters of the AR process, it is possible to estimate parameters that change over time as long as they change more slowly than the (stochastic) dynamic itself. Moreover, the state space model allows one to account for observational noise. Using the Expectation-Maximization algorithm applying the dual Kalman filter, parameters can thus be estimated in a time-resolved manner in the presence of observational noise^9^. A multivariate generalization of the AR model enables the analysis of interdependency between EEG signals.

### Functional and effective connectivity

Functional connectivity estimates typically use FFT-based cross-spectra. The strength of connectivity can be determined by calculating the correlation (coherence) between corresponding components in each signal^6,10^. A significant correlation indicates synchronicity between neuronal populations which implie*s* communication^11^. However, this approach cannot establish the direction of information transfer and differentiate direct from indirect influences between regions.

While brain signals fall into the class of non-linear stochastic signals, stochastic linear modelling exploiting AR processes has been shown to be suitable to infer their key characteristics and interactions^12^. In a recent publication^13^, a unique overarching framework was introduced providing the mathematical foundation explaining the good performance of autoregressive models even when applied to non-linear systems.

Partial directed coherence (PDC) is a measure for Granger-causality^8^ in the frequency domain based on these AR models. Here, we used renormalized PDC (rPDC) which utilizes multivariate AR processes^12,14^, and allows for interpretation of connectivity direction and strength^12^. rPDC overcomes the suboptimal normalisation of PDC by removing the frequency dependence of the confidence intervals. Fully time-resolved parameters can be estimated by using the aforementioned state space model^9^ as follows:

1 $$a(i)=a(i-1)+{\varepsilon }_{a}(t),\,\text{with}\,{\varepsilon }_{a}(t) \sim N(0,{{\rm{\Sigma }}}_{a})$$

2 $$Z(i)=A(i)Z(i-1)+\varepsilon (t),\,{\rm{with}}\,{\rm{\varepsilon }}(t) \sim N(0,{\rm{\Sigma }})$$

3 $$Y(i)=CZ(i)+{\varepsilon }_{Y}(t),\,{\rm{with}}\,{\varepsilon }_{Y}(t) \sim N(0,{{\rm{\Sigma }}}_{Y})$$

where Y(i) is a vector of the observed time series and *ε*_*Y*_(*t*) models the observational noise. The second equation describes the AR process. The a(i) are the matrix entries of A(i) rearranged into a vector. The matrix A(i) contains the interactions between the components of the AR process Z(t). The information about the network structure is therefore contained in this matrix. Time-resolved Granger-causality can be derived from the estimated parameters A(i) from which rPDC is calculated. However, while this has been validated previously based on simulated data^9^, its applicability to “real-world” quantitative EEG (qEEG) data remained to be confirmed.

Validation of qEEG methods based on actual observed data is inherently more difficult than for simulated data as there is no known ground truth against which to compare results and performance. Therefore, we here first compare the novel approaches with their classical counterparts (i.e., AR to FFT and rPDC to coherence). We demonstrate that AR and FFT are equivalent for a chosen experimental example (age-related qEEG changes in mice) and confirm that the additional directional information provided by rPDC is congruous with results obtained via classical coherence.

We then probe the utility of AR-based methods, first in a preclinical application which does not require temporal resolution (drug effects on NREM sleep) followed by an example requiring high temporal resolution (spatial decision making). In the latter case, we demonstrate that time resolved rPDC can capture brief, behaviourally relevant connectivity patterns which underlie spontaneous alternation behaviour.

## Methods

### EEG surgeries

Animals were housed and tested in accordance with European (FELASA) and UK Home Office regulations, experiments were approved by the university’s Ethics Board and carried out in accordance with the Animal (Scientific Procedures) Act 1986. Electrode implantation surgery was conducted as described previously^15^. Briefly, general anaesthesia was induced using inhaled isoflurane and adequate anaesthetic depth was confirmed by lack of limb withdrawal response to interdigital pinch. In all experiments, gold screw electrodes were skull implanted into burr holes drilled in the parietal plate bilaterally above the dorsal hippocampi (2 mm posterior/1.5 mm lateral (right + left) of Bregma) and into the frontal plate above the right prefrontal cortex (2 mm anterior to Bregma immediately lateral to midline). In Experiment 2 an additional recording electrode was placed above the left somatosensory cortex (SSC; 0.2 mm posterior to Bregma, 3.3 mm lateral to midline). Experiment 3 utilized four recording sites, i.e. bilateral for both prefrontal cortex and parietal/hippocampal sites (coordinates as above). Reference and ground electrodes were placed at neutral locations superficial to parietal and occipital cortices. Subcutaneous buprenorphine hydrochloride was administered for pain management prior to withdrawal of anaesthesia. Animals were then allowed 2 weeks convalescence prior to testing.

EEG recordings were obtained under freely moving conditions using wireless data logging devices, i.e. either the Neurologger (New Behaviour, CH; refs^15,16^) or a neural activity tracker (NAT-1) device (Cybula, UK; ref.^17^). The Neurologger (Experiment 1) has an AC input range of ±750 μV, and input is band-pass limited (1–70 Hz); data were sampled at 200 Hz. The NAT-1 device has an AC input range (configurable) of ±1.75 mV, the input is band pass limited (0.3–500 Hz), and data were sampled at 1000 Hz in Experiment 2 and 200 Hz in Experiment 3. Attachment of the device was conducted 1 hour prior to testing to mitigate handling stress and allow the animal to appropriately habituate to the device.

### EEG data analysis

Power Spectra of individual channels were estimated in two different ways:

1. The “traditional” Fast Fourier Transform (FFT) based estimation was run in MatLab 2015a and used to determine the spectrum via smoothing of the periodogram as described in^18^. A triangular window with a smoothing width of h = 1 Hz was chosen to obtain a consistent estimator.
2. The second spectral estimator was based on autoregressive (AR) processes^19^. Briefly, an AR model is fitted to the data and from its coefficients, the respective spectrum is calculated. The model order used here was set as one quarter of sampling rate i.e. p = 50 samples (for data sampled at 200 Hz) and p = 250 samples (for data sampled at 1 kHz). In both cases, the model order therefore corresponds to 250 ms.

Coherence was also determined based on:

1. A “classical” coherence approach, i.e. cross-periodograms were calculated and smoothed (smoothing width = 1 Hz), and
2. A multivariate autoregressive (AR) process to estimate directed Granger causality between data, yielding renormalized partial directed coherence (rPDC) as described in^12^. For rPDC estimation, we again used a model order of sampling rate/4. A combination of rPDC and state space modelling allows a time and frequency-resolved measure for Granger causality^14^.

### Statistical analysis

EEG Frequency band definitions were Delta (1–5 Hz), Theta (5–9 Hz), Alpha (9–14 Hz), Beta (14–20 Hz) and Gamma (20–50 Hz). Statistical comparison of FFT and AR based power spectra between groups/conditions were conducted via non-matching or repeated measures 2-way ANOVAs as appropriate. For within subject comparisons, mean band power (within subject average of power measured across frequency points in band) was also compared using paired 2-tailed t-tests. Differences are reported here for both methods (α = 0.95).

Group/condition differences for coherence and rPDC were analysed for the same bands based on the respective distributions under the alternative. For rPDC this is a chi-square distribution with two degrees of freedom. For a group of *n* independent measurements the sum of the rPDCs is thus chi-square distributed with 2*n degrees of freedom. We used this distribution to generate confidence intervals for each group.

In the case of coherence, the distribution is more complex. However, it was shown^20^ that tanh^−1^[*Coh*(*ω*)] is approximately normally distributed. The mean coherence for a group of n independent measurements is therefore also approximately normally distributed. From this distribution confidence intervals for the mean coherence of each group can be derived. For coherence as well as rPDC, based on the confidence intervals for two groups, we can then check for a significant difference by counting at how many frequencies the results for the two groups overlap. If this number is smaller than what is expected at random, we conclude that a significant difference exists. Thresholds for significance were defined as α = 0.95 both for classical coherence analysis and for rPDC.

For time-resolved rPDC, differences between groups were analysed based on the mean of each group. Standard error of the mean (SEM) defines a confidence interval for each group. Differences between groups of more than one sigma are investigated.

### Experiment 1: Comparison of FFT and AR based methods

Male wild type (PLB1_WT_) mice^16^ were surgically implanted at ~3 months of age. Briefly, 24 hour EEG recordings were conducted at 5 and 13 months of age with animals individually housed in Pheno Typer home cage observation systems (Noldus IT, NL) for the duration of recording. Automated sleep staging of recorded EEG with post hoc manual correction was then carried out using SleepSign (Kissei Comtec Co. Ltd, JP) to identify periods of wakefulness, as well as REM and NREM sleep. Secondary confirmation of wakefulness was provided through detection of movement by the device’s on-board accelerometer. For this study, 6 mice from each age group were selected for analysis based on optimal quality of EEG recordings in all channels. A 100 second artefact-free EEG strip (during wakefulness) was extracted for each animal. Power spectra were then calculated using both FFT and AR analysis and pooled by age group. Cross-channel coherence was calculated using both classical and rPDC methods.

### Experiment 2: Drug effects on AR spectra and rPDC

Ten male PLB1_WT_ mice were implanted at ~4 months of age to investigate the effects of ketamine and diazepam on sleep and EEG profiles. A 4-week Latin square based drug administration regime was constructed where animals were allocated to 4 groups. Once per week each group received an intraperitoneal injection of either 10 mg/kg ketamine hydrochloride (Tocris Bioscience, UK) in vehicle (saline), saline only (ketamine vehicle), diazepam (Sigma Aldrich, UK) in a vehicle composed of a 1:1:18 ratio of ethanol, Cremaphor and saline, or the diazepam vehicle only. Treatment group allocations were rotated such that each animal received each of the 4 treatments with a 1 week washout between treatments. Following administration, recording devices were attached and animals were placed in individual holding cages where EEG activity was recorded for 6 hours. Five animals were selected for data analysis where high quality recordings had been obtained from recordings conducted both after administration of a drug treatment and administration of its respective vehicle. Sleep staging of EEG was conducted as in experiment 1. Here, a 60 second artefact free strip of EEG from periods of NREM sleep was extracted for each animal and condition. Power spectra and cross-channel coherence were then assessed using AR and rPDC analyses, respectively, and results were pooled by treatment group.

### Experiment 3: Time resolved rPDC analysis of spontaneous alternation in the Y-Maze

Five female PLB1_WT_ mice were implanted at 6 months of age for Y-maze behavioural testing. As in our previous investigations^21^ the Y-maze was constructed from white acrylic and consisted of 3 open topped corridors (arms), each 60 cm long, meeting at a triangular intersection termed ‘the central zone’ (CZ). Each arm (labelled A, B and C) was subdivided into an inner (I) zone (30 cm proximal to CZ) and an outer (O) zone (30 cm distal to CZ). The demarcation of these zones is presented in Suppl. Fig. 1a. At the start of each trial the animal was placed at the distal end of arm B (BO) facing toward the CZ. Upon release, the motion tracking software was activated and the animal was allowed to freely explore the maze for 10 minutes. Transitions between maze arms during the trial were recorded both automatically by the software and manually by an observer viewing the trial on a monitor in a segregated cubicle. The animal was considered to have entered an arm when all 4 paws had moved from the CZ to one of the inner zones.

Transitions between arms (with the exception of the first two) were scored as either correct or incorrect alternations by the observer. A correct alternation was defined by a triplet of arm entries where the animal visited all 3 arms in sequence without repetition (ABC, BCA etc.). An incorrect alternation (or alternate arm re-entries) was defined by a triplet where the first and third entry were the same arm (ABA, BCB etc.). A third category, termed direct repeats, was defined as a triplet which contained one or more consecutive entries into the same arm (AAB, BAA etc.).

Consistent with expectations, correct alternations were the most common triplet type accounting for 58.69% of triplets (SEM = 2.95), followed by incorrect alternations (34.49%, SEM = 4.44) and direct repeats (6.81% SEM = 3.30). Due to the rarity of direct repeats (absent in 2 animals) they were not considered for EEG analysis.

### Motion tracking and event time stamping

Animal movement was recorded by a ceiling mounted camera offering a planar view of the maze. Camera output was digitized using a RTV-24 frame grabber PCI card (ADLINK Technology Inc.). Motion was tracked by a PC running the ANYMAZE 4.9 (Ugo Basile Srl, IT) video tracking package. ANYMAZE was configured such that transitions between zones resulted in the generation of TTL level output trigger pulses unique for the 7 zones of the maze. These pulses were sent to an Arduino Uno rev.2 micro-controller which triggered a unique sequence of on-off pulses to drive flashes from an infra-red (IR) LED array via a MOSFET driver. These IR flashes were detected by an IR receiver module on the NAT-1 device and used to time stamp zone transition events onto the EEG signal.

### Extraction of behaviourally relevant EEG segments

Data from the NAT-1 devices (4 EEG channels and IR channel) were downloaded as CSV files. Time resolved coordinates representing the animal’s location were exported from ANYMAZE (also in CSV format) along with TTL time stamps to spatially and temporally synchronize data, i.e. the animal’s location and the EEG time series. This procedure was accomplished using an in-house written MATLAB (MathWorks Inc, USA) script. Six second segments of EEG data corresponding to either correct or incorrect transitions (see above) were identified and extracted for analysis.

To control for gross differences in movement speed and location, strips were taken from arm transitions where the animal had moved from the outer extremity of one arm to the outer extremity of another during the extraction time frame. This step was taken to ensure that the motion paths of animals were comparable for all EEG segments analysed. Transitions (3 correct and 3 incorrect per animal) were selected from each animal (n = 5) for analysis. Data strips were balanced for turn direction (i.e. 6 of the 15 samples representing correct transitions were right hand turns and 9 were left; incorrect samples were composed of 7 right and 8 left hand turns). Exemplar motion paths of animals during the analysis time frame are presented in Suppl. Figure 1b. The position of animals relative to the centre of the maze over the 6 seconds of data extracted is presented in Suppl. Figure 1c.

AR power spectra for each alternation type were calculated, averaged within subjects and subsequently pooled. An rPDC analysis for the above data set was conducted using both time stationary and time resolved techniques. For the time resolved analysis, rPDC strength was calculated, resolved in both time and frequency for all 30 EEG samples. Results were pooled to calculate the population median coherence strength observed over all channel pairs. The data were then partitioned into correct and incorrect alternations and grouped heat plots created to provide a visual comparison of changes in coherence strength between alternation types. The population median coherence value was used as a colour scale normalization factor to ensure that grouped heat plots of correct and incorrect alternations were directly comparable.

## Results

### Experiment 1: Effect of age in wild-type mice

#### Regional analysis: FFT vs AR Spectra

**Figure 1:**
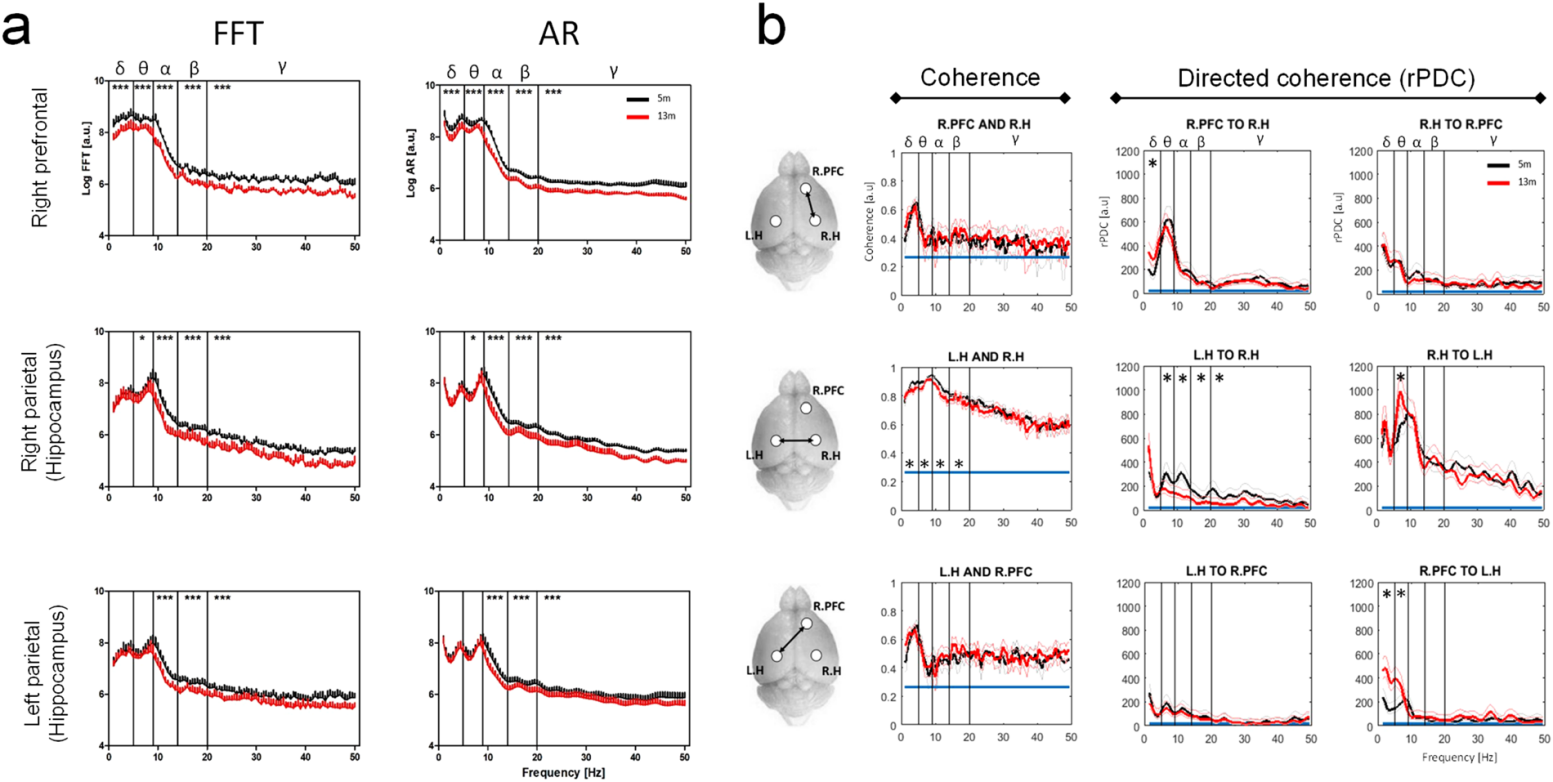
FFT vs AR power spectra & coherence vs rPDC: Effect of age on EEG power and connectivity in WT mice. (**a**) EEG power (as log arbitrary units [a.u.], means + SEM) illustrated over the 1–50 Hz range for mice aged 5 m (black) and 13 m (red). Left: FFT-based power spectra for each recording site, right: corresponding spectra calculated using autoregressive spectral estimation (AR). Asterisks on plots provide a p-value summary for the effect of age (*p < 0.05, **p < 0.01, ***p < 0.001). (**b**) Mean “classical” coherence strength [a.u., left column] observed between all possible channel pairs over the 1–50 Hz range for mice aged 5 m (black) and 13 m (red). Corresponding rPDC strength [a.u.] over the same frequency range for all possible channel pairs and directions of coherence are depicted in the middle and right columns. Pictograms on the left illustrate which channel pairs are analysed in each row. Plot titles indicate compared channels and, where applicable, direction of coherence. R.PFC = right prefrontal, R.H = right parietal (hippocampus), L.H = left parietal (hippocampus). Blue lines = 95% confidence limit. Lightly coloured lines indicate the confidence interval of the group means. Significant differences between age groups are illustrated for individual frequency bands (*p < 0.05).

Power spectra from 100 second EEG strips were calculated using both FFT and AR based methods and compared between young (5 m) and aged (13 m) wild-type mice (PLB1_WT_) (Fig. 1a & Table 1). Visual inspection indicates an improved smoothness (and hence identification of peaks) of AR vs. FFT spectra, likely due the reduced impact of noise. However, for the purpose of quantitative analysis, both methods were statistically equivalent, and detected global reductions in power with age for most frequency bands. These findings confirmed that AR modelling results in accurate reconstruction of the underlying power spectrum and detection of age related changes.

**Table 1:** Summary and comparison for the effect of age (FFT vs AR power spectra).

Right prefrontal		F		P	
Frequency band	DF (Age, Residual)	FFT	AR	FFT	AR
**Right parietal (Hippocampus)**		**F**		**P**	
**Frequency band**	**DF (Age, Residual)**	**FFT**	**AR**	**FFT**	**AR**
**Left parietal (Hippocampus)**		**F**		**P**	
**Frequency band**	**DF (Age, Residual)**	**FFT**	**AR**	**FFT**	**AR**
Delta (1–5 Hz)	1,90	21.86	18.67	<0.0001	<0.0001
Theta (5–9 Hz)	1,90	22.72	21.33	<0.0001	<0.0001
Alpha (9–14 Hz)	1,110	88.98	96.12	<0.0001	<0.0001
Beta (14–20 Hz)	1,120	65.29	91.95	<0.0001	<0.0001
Gamma (20–50 Hz)	1,610	464.7	482.1	<0.0001	<0.0001
Delta (1–5 Hz)	1,90	2.101	1.424	0.1507	0.2359
Theta (5–9 Hz)	1,90	6.397	5.019	0.0132	0.0275
Alpha (9–14 Hz)	1,110	36.25	32.49	<0.0001	<0.0001
Beta (14–20 Hz)	1,120	30.09	33.6	<0.0001	<0.0001
Gamma (20–50 Hz)	1,610	231.7	287.6	<0.0001	<0.0001
Delta (1–5 Hz)	1,90	<1	<1	0.4263	0.7773
Theta (5–9 Hz)	1,90	2.365	1.261	0.1276	0.2644
Alpha (9–14 Hz)	1,110	24.99	19.75	<0.0001	<0.0001
Beta (14–20 Hz)	1,120	27.12	17.21	<0.0001	<0.0001
Gamma (20–50 Hz)	1,610	100.2	47.14	<0.0001	<0.0001

Overview for the effect of age under 2-way ANOVAs comparing EEG power spectral density of mice aged 5 and 13 months for key frequency bands. Degrees of freedom (DF) as well as P and F values are provided for comparisons of both FFT and AR power spectra.

#### Connectivity: Classical coherence vs rPDC

Connectivity measures were compared between young and aged mice for all possible channel pairs using both ‘classical’ coherence and rPDC (Fig. 1b). The former confirmed robust connectivity between all channel pairings for both groups and an effect of age regarding a reduction intra-hippocampal connectivity in all frequency bands except gamma. By contrast, a number of additional age-related effects were detected using rPDC: left to right hippocampal connectivity was reduced in all frequency bands except Delta, while Theta coherence between these channels was *enhanced* in the opposite direction. Enhanced Theta coherence was also observed in aged mice for more complex connections (i.e. right pre-frontal cortex to the contralateral hippocampus) as well as enhanced Delta coherence from the right prefrontal cortex to both hippocampi.

### Experiment 2: Effects of diazepam and ketamine

#### Drug effects on vigilance stages

We next assessed the actions of diazepam (2 mg/Kg) and ketamine (10 mg/Kg) on NREM sleep in PLB1_WT_ mice. Diazepam decreased [t = 4.961, df = 4, p < 0.01] while ketamine increased onset to sleep [t = 3.816, df = 4, p < 0.05], (Suppl. Figure 2). In the 6 hrs following drug injection, diazepam increased NREM sleep and reduced waking activity, while opposing trends were seen in ketamine treated mice.

#### Diazepam reduced NREM Delta/Theta power and increased anterior Gamma power

**Figure 2:**
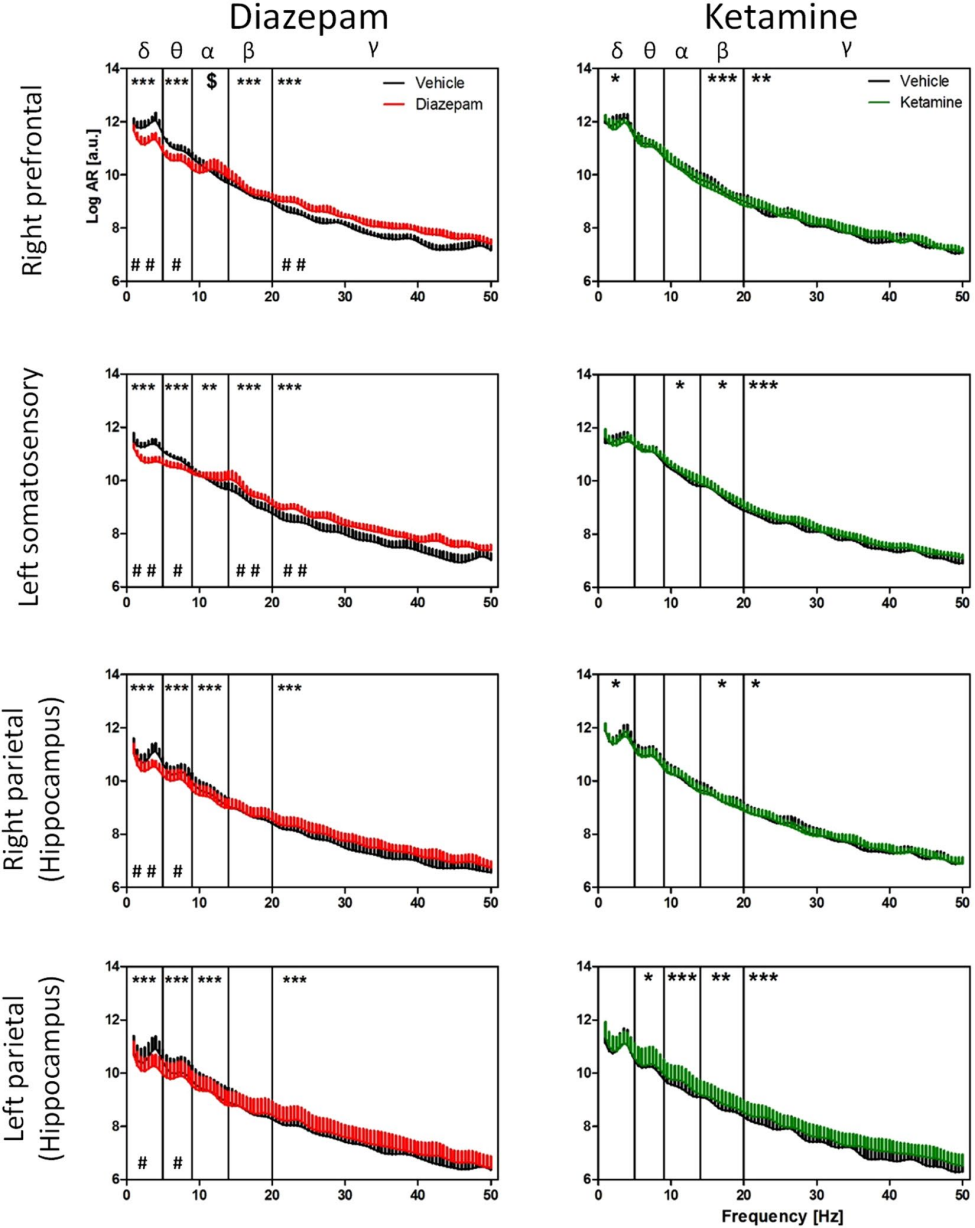
Effect of diazepam and ketamine on EEG power. AR power spectra (as log arbitrary units [a.u.], +SEM) during NREM sleep over the 1–50 Hz frequency range for mice treated with 2 mg/kg diazepam (red) or 10 mg/kg ketamine (green) vs mice treated with vehicle only (black). Effect of drug treatment is indicated as *p < 0.05, **p < 0.01, ***p < 0.001. Hash signs illustrate overall band power differences between treatments (^#^p < 0.05, ^##^p < 0.01).

NREM AR power spectra obtained from diazepam/ketamine treated mice alongside respective vehicle controls (Fig. 2 & Tables 2–3) suggest that diazepam significantly *reduced* NREM Delta and Theta power relative to vehicle controls in all 4 recording sites, while Gamma power in the right prefrontal cortex as well as Beta and Gamma power in the left somatosensory cortex increased significantly. Bilaterally increased Gamma band power was also indicated in the parietal recording sites, this was not confirmed for mean band power. By contrast, despite multiple significant effects of ketamine on frequency-resolved bands, no differences were detected for mean band power.

**Table 2:** Effect of drugs on NREM AR power spectra.

Right prefrontal		Diazepam		Ketamine	
Frequency band	DF (Drug, Residual)	F	P	F	P
**Right somatosensory**		**Diazepam**		**Ketamine**	
**Frequency band**	**DF (Drug, Residual)**	**F**	**P**	**F**	**P**
**Right parietal (Hippocampus)**		**Diazepam**		**Ketamine**	
**Frequency band**	**DF (Drug, Residual)**	**F**	**P**	**F**	**P**
**Left parietal (Hippocampus)**		**Diazepam**		**Ketamine**	
**Frequency band**	**DF (Drug, Residual)**	**F**	**P**	**F**	**P**
Delta (1–5 Hz)	1,36	104	<0.0001	5.268	0.0276
Theta (5–9 Hz)	1,36	107.5	<0.0001	2.736	0.1068
Alpha (9–14 Hz)	1,44	<1	0.7123^**$**^	2.985	0.091
Beta (14–20 Hz)	1,52	30.62	<0.0001	27.15	<0.0001
Gamma (20–50 Hz)	1,244	708.7	<0.0001	6.903	0.0091
Delta (1–5 Hz)	1,36	135	<0.0001	2.831	0.1011
Theta (5–9 Hz)	1,36	48.92	<0.0001	<1	0.9285
Alpha (9–14 Hz)	1,44	7.572	0.0086	5.505	0.0235
Beta (14–20 Hz)	1,52	149.4	<0.0001	6.373	0.0147
Gamma (20–50 Hz)	1,244	864.5	<0.0001	50.32	<0.0001
Delta (1–5 Hz)	1,36	137.4	<0.0001	5.491	0.0248
Theta (5–9 Hz)	1,36	47.58	<0.0001	4.02	0.0525
Alpha (9–14 Hz)	1,44	33.85	<0.0001	<1	0.9718
Beta (14–20 Hz)	1,52	2.055	0.1577	5.079	0.0285
Gamma (20–50 Hz)	**1,244**	**152.5**	**<0.0001**	**4.346**	**0.0381**
Delta (1–5 Hz)	1,36	84.14	<0.0001	1.381	0.2476
Theta (5–9 Hz)	1,36	32.47	<0.0001	4.244	0.0467
Alpha (9–14 Hz)	1,44	17.69	0.0001	15.01	0.0004
Beta (14–20 Hz)	1,52	2.648	0.1097	10.6	0.002
Gamma (20–50 Hz)	1,244	136.8	<0.0001	94.51	<0.0001

Results are provided for both diazepam and ketamine effects (repeated measures 2-way ANOVAs) comparing EEG power spectral density of mice under drug treatment with those of the same mice treated with corresponding vehicles. Degrees of freedom (DF) as well as P and F values are provided for each drug treatment. Dollar signs indicate that a significant drug/frequency interaction effect was observed in the absence of significant overall effect of drug. ^$^p<0.05.

**Table 3:** Effect of drugs on NREM mean AR band power.

Right prefrontal	Diazepam			Ketamine		
Frequency band	T	DF	P	T	DF	P
**Left somatosensory**	**Diazepam**			**Ketamine**		
**Frequency band**	**T**	**DF**	**P**	**T**	**DF**	**P**
**Right parietal (Hippocampus)**	**Diazepam**			**Ketamine**		
**Frequency band**	**T**	**DF**	**P**	**T**	**DF**	**P**
**Left parietal (Hippocampus)**	**Diazepam**			**Ketamine**		
**Frequency band**	**T**	**DF**	**P**	**T**	**DF**	**P**
Delta (1–5 Hz)	4.967	4	0.0077	1.103	4	0.3321
Theta (5–9 Hz)	3.777	4	0.0195	<1	4	0.588
Alpha (9–14 Hz)	<1	4	0.9101	<1	4	0.6043
Beta (14–20 Hz)	2.175	4	0.0953	1.601	4	0.1846
Gamma (20–50 Hz)	8.348	4	0.0011	<1	4	0.6514
Delta (1–5 Hz)	4.995	4	0.0075	<1	4	0.4676
Theta (5–9 Hz)	3.196	4	0.033	<1	4	0.9737
Alpha (9–14 Hz)	1.332	4	0.2537	<1	4	0.4939
Beta (14–20 Hz)	6.679	4	0.0026	<1	4	0.5065
Gamma (20–50 Hz)	4.716	4	0.0092	1.022	4	0.3647
Delta (1–5 Hz)	6.939	4	0.0023	1.065	4	0.3469
Theta (5–9 Hz)	3.906	4	0.0174	<1	4	0.4753
Alpha (9–14 Hz)	1.939	4	0.1246	<1	4	0.991
Beta (14–20 Hz)	<1	4	0.6907	<1	4	0.4033
Gamma (20–50 Hz)	1.844	4	0.139	<1	4	0.6324
Delta (1–5 Hz)	4.197	4	0.0137	<1	4	0.663
Theta (5–9 Hz)	3.490	4	0.0251	<1	4	0.519
Alpha (9–14 Hz)	1.535	4	0.1996	<1	4	0.3005
Beta (14–20 Hz)	<1	4	0.587	<1	4	0.406
Gamma (20–50 Hz)	1.758	4	0.1535	1.353	4	0.2475

Summary of mean band power comparison (paired 2-tailed t-tests) of mice treated with either diazepam or ketamine cf corresponding vehicle groups. Degrees of freedom (DF) as well as T and F values are provided for each drug treatment.

#### Diazepam decreased, while ketamine increased, prefrontal - hippocampal functional connectivity

**Figure 3:**
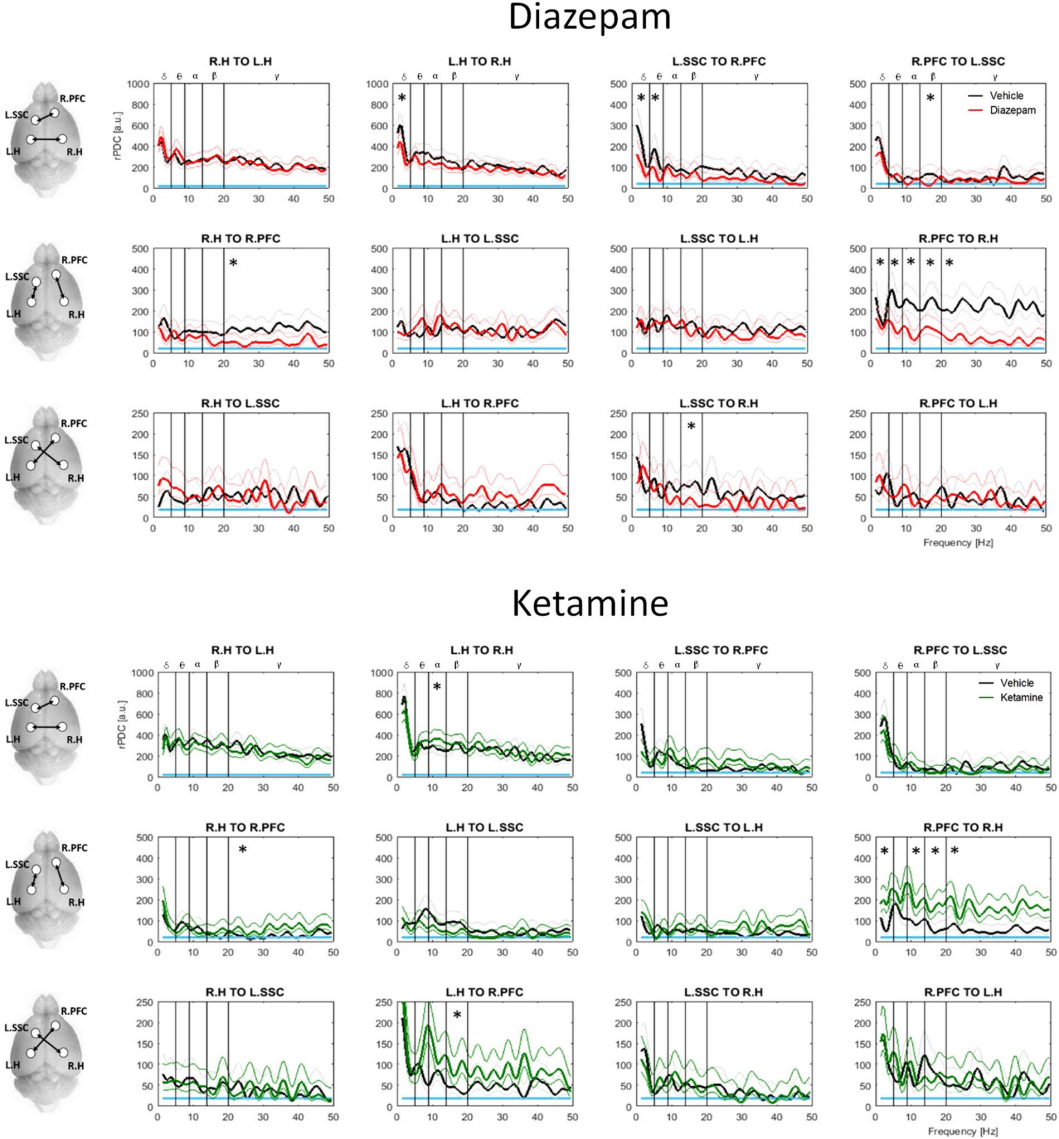
Differential effects of Diazepam and Ketamine on connectivity (rPDC) in NREM sleep. Mean coherence (rPDC) strength is shown in arbitrary units [a.u.] as observed between all possible channel pairs over the 1–50 Hz range for mice treated with diazepam (red) or ketamine (green) relative to respective vehicles (black). Pictograms to the left of plots indicate channel pairs analysed in each row. Subplot titles indicate channels and direction of coherence; R.PFC = right prefrontal, R.H = right parietal (hippocampus), L.H = left parietal (hippocampus) L.SSC = left somatosensory. Blue lines = 95% confidence limit. Lightly coloured lines = confidence interval of group means. Significant drug effects are illustrated for individual frequency bands (*p < 0.05).

Drug effects on connectivity (rPDC; Fig. 3) suggested a significant reduction in coherence strength for diazepam vs vehicle treatment. In direct contrast, ketamine exclusively increased interregional coherence. This difference was most striking in communication from the right prefrontal cortex to right hippocampus where diazepam reduced coherence across all frequency bands while ketamine increased coherence in all bands except Theta. A similar contrast was observed in Gamma coherence between the same channels in the opposing direction. Ketamine also significantly increased Beta coherence from the left hippocampus to the right prefrontal cortex, and Alpha coherence from the left-to-right hippocampus.

Additional band specific reductions in connectivity were also observed under diazepam. Diazepam decreased Delta coherence from the left-to-right hippocampus, Delta  and Theta coherence from the somatosensory to the right prefrontal cortex (and Beta coherence in the opposing direction), as well as Beta coherence from the left somatosensory cortex to the right hippocampus.

For methodological comparison, classical coherence analysis was also performed (Suppl. Figure 3). Only one significant drug effect was noted, an elevated theta coherence between the left hippocampus and somatosensory cortex under diazepam. Interestingly, no significant drug effects emerged in this range between these channels based on rPDC. A trend toward elevated theta coherence under diazepam was however noted in the left hippocampus to somatosensory cortex direction (Fig. 3).

### Experiment 3: AR and rPDC analysis of brain activity during spontaneous alternation

#### AR spectra during correct vs. incorrect alternations

**Figure 4:**
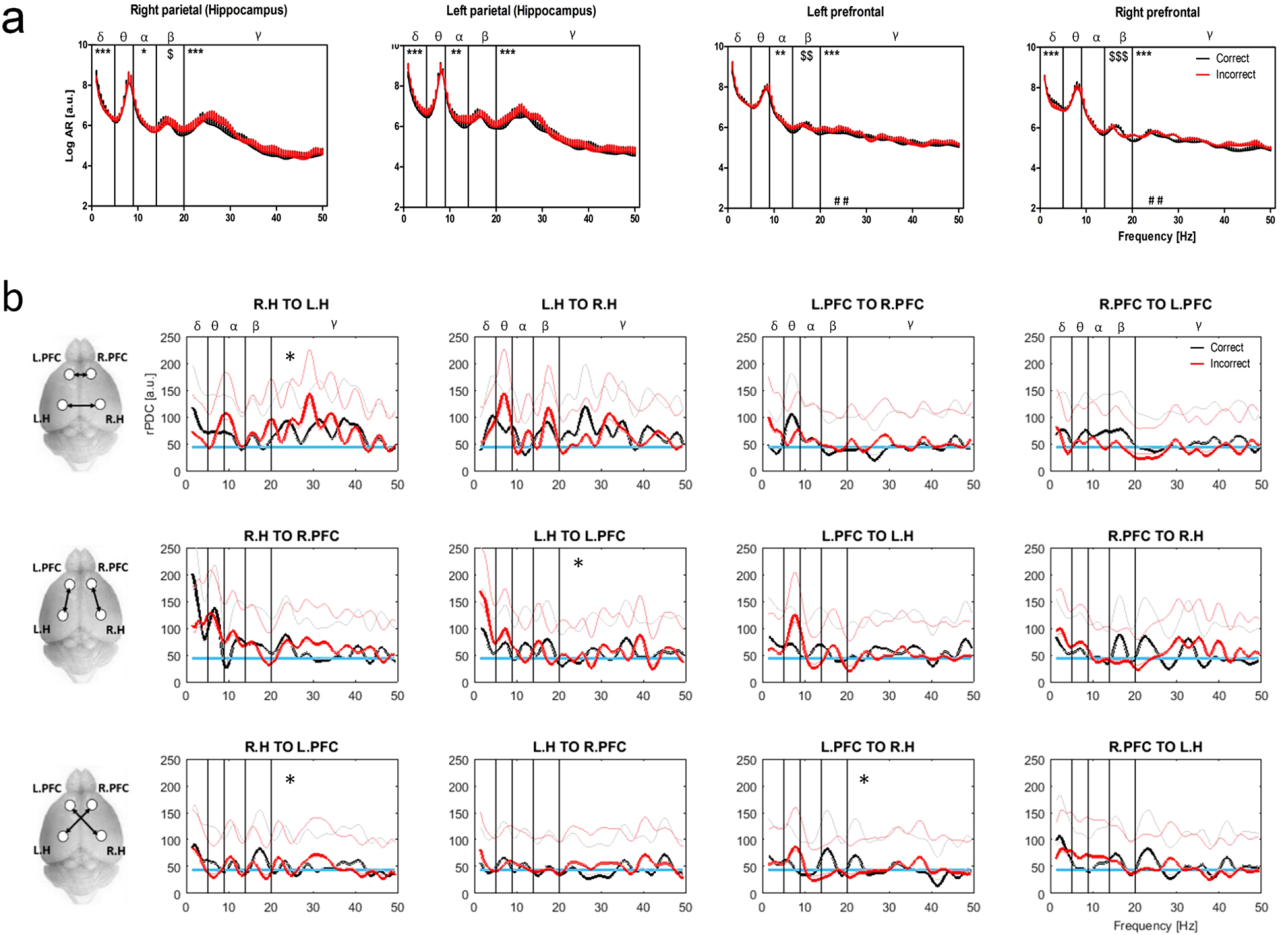
Autoregressive power spectra and connectivity (rPDC) during correct and incorrect Y-maze alternations. (**a**) AR spectral power (in log arbitrary units [a.u.], + SEM) over the 1–50 Hz frequency range during correct (black) and incorrect (red) alternations in the Y-maze. Data were obtained from 3 correct and 3 incorrect EEG strips per animal and averaged within subjects (n = 5). Effect of alternation type *p < 0.05, **p < 0.01, ***p < 0.001. Dollar signs indicate significant alternation type/frequency interaction where there is no significant overall effect of alternation (^$^p < 0.05, ^$$^p < 0.01, ^$$$^p < 0.001). Hash signs indicate significant differences in mean band power (^##^p < 0.01). (**b**) Mean coherence (rPDC) strength [a.u.] observed between all possible channel pairs over the 1–50 Hz range during correct (black) and incorrect (red) alternations (15 samples per condition). Pictograms (left) illustrate channel pairs analysed in each row. Subplot titles indicate channel pair and direction of coherence; R.PFC = right prefrontal, L.PFC = left prefrontal, R.H = right parietal (hippocampus), L.H = left parietal (hippocampus). Blue lines = 95% confidence limit. Faintly coloured lines give confidence interval of group means. Asterisks indicate significant differences between alternation types.

AR power spectra were calculated for 15 samples of EEG capturing correct and 15 capturing incorrect Y-maze alternations then averaged within subjects (n = 5; Fig. 4a & Tables 4, 5). A significantly higher prefrontal Gamma power was observed in incorrect vs correct alternations. A similar trend was observed in the right hippocampal recording site. Significant differences at lower frequencies were indicated for all channels, however these were not corroborated by mean band power analysis.

**Table 4:** Effect of Y-Maze alternation type on power spectra.

Right prefrontal			
Frequency band	DF (Alt type, Residual)	F	P
**Left prefrontal**			
**Frequency band**	**DF (Alt type, Residual)**	**F**	**P**
**Right parietal (Hippocampus)**			
**Frequency band**	**DF (Alt type, Residual)**	**F**	**P**
**Left parietal (Hippocampus)**			
**Frequency band**	**DF (Alt type, Residual)**	**F**	**P**
Delta (1–5 Hz)	1,36	14.72	0.0005
Theta (5–9 Hz)	1,36	1.532	0.2238
Alpha (9–14 Hz)	1,44	1.189	0.2815
Beta (14–20 Hz)	1,52	<1	0.8692^$$$^
Gamma (20–50 Hz)	1,244	103.8	<0.0001
Delta (1–5 Hz)	1,36	2.04	0.1618
Theta (5–9 Hz)	1,36	<1	0.7061
Alpha (9–14 Hz)	1,44	8.891	0.0047
Beta (14–20 Hz)	1,52	2.386	0.1285^$$^
Gamma (20–50 Hz)	1,244	29.02	<0.0001
Delta (1–5 Hz)	1,36	23.04	<0.0001
Theta (5–9 Hz)	1,36	<1	0.4011
Alpha (9–14 Hz)	1,44	4.912	0.0319
Beta (14–20 Hz)	1,52	<1	0.345^$^
Gamma (20–50 Hz)	1,244	32.76	<0.0001
Delta (1–5 Hz)	1,36	32.41	<0.0001
Theta (5–9 Hz)	1,36	2.061	0.1598
Alpha (9–14 Hz)	1,44	10.54	0.0022
Beta (14–20 Hz)	1,52	1.593	0.2126
Gamma (20–50 Hz)	1,244	26.69	<0.0001

Effect of correct vs incorrect alternations (‘Alt type’) comparing EEG power in the Y-Maze (repeated measures 2-way ANOVA). Degrees of freedom (DF) as well as P and F values are provided for each EEG band. Dollar signs indicate significant interactions (alternation type × frequency) in the absence of significant overall drug effect ^$^p < 0.05, ^$$^p < 0.01, ^$$$^p < 0.001.

**Table 5:** Effect of alternation type on EEG band power.

Right prefrontal			
Frequency band	T	DF	P
**Left prefrontal**			
**Frequency band**	**T**	**DF**	**P**
**Right parietal (Hippocampus)**			
**Frequency band**	**T**	**DF**	**P**
**Right parietal (Hippocampus)**			
**Frequency band**	**T**	**DF**	**P**
Delta (1–5 Hz)	1.656	4	0.173
Theta (5–9 Hz)	<1	4	0.5341
Alpha (9–14 Hz)	<1	4	0.6841
Beta (14–20 Hz)	<1	4	0.9391
Gamma (20–50 Hz)	4.877	4	0.0082
Delta (1–5 Hz)	<1	4	0.5377
Theta (5–9 Hz)	<1	4	0.8586
Alpha (9–14 Hz)	1.084	4	0.3392
Beta (14–20 Hz)	<1	4	0.5619
Gamma (20–50 Hz)	7.280	4	0.0019
Delta (1–5 Hz)	2.203	4	0.0923
Theta (5–9 Hz)	<1	4	0.6059
Alpha (9–14 Hz)	<1	4	0.3742
Beta (14–20 Hz)	<1	4	0.713
Gamma (20–50 Hz)	2.445	4	0.0708
Delta (1–5 Hz)	2.100	4	0.1036
Theta (5–9 Hz)	<1	4	0.5569
Alpha (9–14 Hz)	1.363	4	0.2446
Beta (14–20 Hz)	<1	4	0.5001
Gamma (20–50 Hz)	1.403	4	0.2332

Summary of paired 2-tailed t-test statistics comparing mean band power of mice during correct and incorrect Y-Maze alternations (within subjects design). Degrees of freedom (DF) as well as T and F values are provided for each EEG band.

#### Directional connectivity (rPDC) during correct and incorrect alternations

Time invariant functional connectivity (rPDC) over the 1–50 Hz range during correct and incorrect alternations in the Y-maze (Fig. 4b) suggest coherence to be highest (particularly in the Delta-Theta range) between left and right hippocampi, and from the hippocampi to the ipsilateral prefrontal cortices. Differences detected between correct and incorrect alternations were entirely confined to the Gamma band, and detected from the right hippocampus to the left hippocampus and left prefrontal cortex, from the left hippocampus to the left prefrontal cortex and from the left prefrontal cortex to the right hippocampus. However, in all cases, Gamma coherence was affected in a bi-directional manner and consequently, lines intersected repeatedly. Additionally, the magnitude of coherence observed in the Gamma range in these analyses was close to (and occasionally below) the 95% confidence limit at multiple frequency points. Therefore, results relating to the Gamma band should be considered with caution.

#### Time resolved effective connectivity during correct vs. incorrect alternations

**Figure 5:**
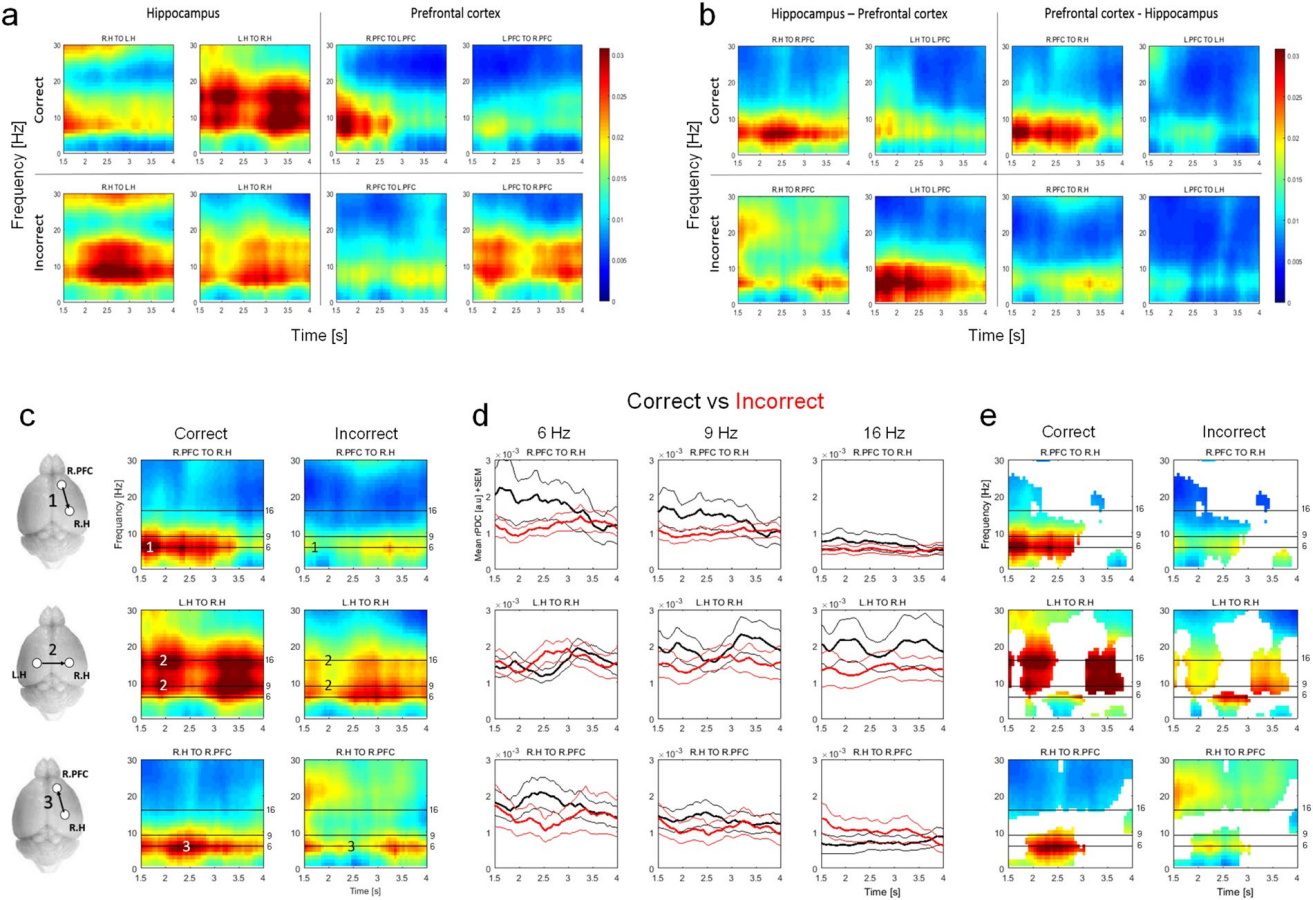
Time resolved connectivity (rPDC) on central zone crossing in the Y-Maze: Correct vs incorrect alternations. Strength of contralateral homotypic (**a**) and ipsilateral heterotypic (**b**) coherence (rPDC) resolved in both time (seconds - X-axis) and frequency (Hz - Y-axis) based on 15 EEG samples capturing correct and 15 incorrect Y-Maze alternations. Colour bars translate rPDC arbitrary units [a.u.] to corresponding colour. Subplot titles indicate channel pair and direction of coherence; left parietal (hippocampus) = L.H, right parietal (hippocampus) = R.H, left prefrontal cortex = L.PFC and right prefrontal cortex = R.PFC. Time window corresponds to transition through central zone of Y-Maze [see Suppl. Fig. 1c]. Correct and incorrect alternation types are stratified by row. (**c**): Isolation of a specific temporal pattern of directed coherence unique to correct alternations. **(c)1:** 6 Hz signal from R.PFC to R.H at time = 1.6 s **(c)2:** 6 and 9 Hz signals from L.H to the R.H at 1.9 s. **(c)3**: 6 Hz signal from R.H to R.PFC from 2–3 s. (**d**) rPDC strength **(**mean ± SEM) at 6, 9 and 16 Hz plotted over time for correct (black) and incorrect (red) alternations. (**e**) Heat plots (as in **c**), modified to only show time-frequency points where rPDC strength differs by more than one sigma between alternation types.

Cognitive processes in a navigating animal must be expected to be highly dynamic. This was indeed demonstrated using heat plots of time-resolved rPDC over the 6 seconds analysed for correct and incorrect alternations (Suppl. Figures 4 and 5). From these plots, the greatest distinction between correct and incorrect alternations emerged in contralateral homotypic (left-to-right hippocampus) and ipsilateral heterotypic (right prefrontal to right hippocampus) directions and in a time window between 1.5 and 4 seconds. This corresponds to the animals’ passage through the central zone of the maze (Suppl. Figure 1c). In order to illustrate these differences, plots of coherence (for the frequency range 1–30 Hz) over the identified time period are presented for interhemispheric homotypic connections in Fig. 5a and for ipsilateral heterotypic connections in Fig. 5b.

Qualitative examination of interhemispheric homotypic coherence during central zone crossings revealed an interesting contrast in the coherence patterns of correct vs. incorrect transitions: Between the hippocampi, directed coherence in the 5–15 Hz range was markedly stronger in the left-to-right direction than right-to-left during correct alternations. During incorrect alternations the same coherence was stronger in the opposing direction. Furthermore, the inverse pattern was seen in the prefrontal cortex: 5–15 Hz coherence was stronger in the right-to-left direction than in the left-to-right during correct alternations. Again, during incorrect alternations the opposite was true. This effect was also visible in plots of ipsilateral heterotypic coherence, i.e. 5–10 Hz coherence from the hippocampus to the prefrontal cortex was strongest in the left hemisphere during correct alternations but in the right hemisphere during incorrect alternations.

Subsequent statistical comparison of time resolved coherence between correct and incorrect alternations revealed multiple time-frequency regions where groups differed in rPDC strength by more than one sigma (see Fig. 5e). Most salient among these was a specific temporal pattern of rPDC (highlighted in Fig. 5c) clearly apparent in correct and absent from incorrect alternations:

1. A burst of right prefrontal to right hippocampal Theta coherence, strongest at ~6 Hz at 1.6–1.8 seconds.
2. Simultaneous bursts of high Theta and Beta coherence from the left to right hippocampus, strongest at ~9 Hz and ~16 Hz, respectively, observed from 1.9 seconds onwards. Similar bursts were also observed ~ 3.1 s in both correct and incorrect alternations but remained stronger in the correct case.
3. A sustained period of Theta coherence from the right hippocampus to right prefrontal cortex at ~6 Hz from 2–3 s. This coincided with the period between intra-hippocampal Theta/Beta coherence bursts described in 2.

The rPDC strength (mean ± SEM) at each of the key frequencies (6, 9 or 16 Hz, indicated by black lines in Fig. 5c and 5e) is illustrated over time for both correct and incorrect alternations to visualize both magnitude of difference and variability (Fig. 5d).

## Discussion

This paper sought to validate improved qEEG algorithms in key neuroscientific applications. Deciphering directional changes as well as direct vs indirect routes of communication between channels was found to be critical for our understanding of network phenomena. Previous attempts to provide time resolution of directional connectivity typically employed non-linear auto-regression based approaches^22–24^. However, these approaches were limited in their temporal resolution and only easily applied to bivariate analyses. We therefore propose that the time-resolved combination of rPDC with state space modelling offers a superior solution, whilst also dealing with non-linear and noisy properties of biopotentials.

Comparison of classical functional connectivity (coherence) with directed functional connectivity (or effective connectivity; rPDC) indicated logical agreements between the methods. For example, a generally high level of coherence was observed between contralateral homotypic sites densely anatomically interconnected by callosal/commissural fibres^25^. Yet, rPDC produced a wealth of additional details and was able to elaborate on hippocampal-prefrontal network communications and their modulation by physiological states. Some connectivity patterns are particularly difficult to resolve with traditional methods, for example, ipsilateral prefrontal-hippocampal coherence, where rPDC indicated significant coherence in both directions. As the prefrontal – hippocampal connection is anatomically indirect^26–28^ communication along this tract can be assumed to be underestimated by classical coherence analysis.

### Effect of age

AR spectra confirmed a global age-dependent reduction of EEG power for all frequencies at prefrontal channels, and at frequencies above Delta in the hippocampal channel. The first salient finding from the rPDC analysis was an increased Delta coherence from the prefrontal cortex to the ipsilateral and contralateral hippocampus. Increased Delta coherence is a frequent observation in human AD and has been associated with cholinergic dysfunction^29,30^. Reports on the effect of normal physiological aging specifically on Delta coherence are however absent from the literature. More generally, interhemispheric coherence between homotypic regions reliably decreases in resting awake subjects across multiple frequency bands with normal aging^31,32^ and is thought to result from loss of callosal projections^31,33^. This is consistent with our observation of reduced left – right hippocampal connectivity in aged mice and with results of classical coherence analysis. However, Theta coherence was found to be enhanced in aged mice in the right-left direction under rPDC. As classical coherence essentially sums coherence in both directions, the opposing Theta changes observed here were likely rendered invisible thus far. Additionally, right-left coherence was markedly stronger in the opposing direction for both age groups. rPDC results therefore indicate that a symmetrical loss of callosal projection fibres between the hippocampi is an incomplete explanation. Rather, age-related degradation of hippocampus dependent memory in rodents^34,35^ may involve both loss of function alongside compensatory increase in specific frequency bands. rPDC can therefore offer new avenues to study hemisphere-specific changes and functional lateralization (e.g., refs^36,37^). Critically, findings provided by rPDC do not violate the predictions of classical coherence nor those of existing literature, i.e. summation of changes in rPDC over both directions would result in the cancellation of opposing changes, and thus similar connectivity estimates to those provided by classical methods.

### Pharmaco-EEG

Spectral analysis indicated that diazepam reduced Delta-Theta activity and enhanced Gamma activity in the prefrontal and somatosensory cortices. This is a known effect of diazepam often termed “the benzodiazepine fingerprint”^38^. By contrast, the anticipated increase in NREM Delta band power under ketamine^39^ was not detected, likely attributable to dosage and timing differences.

Striking differences between the actions of ketamine and diazepam were confirmed via rPDC analysis particually for the ipsilateral prefrontal–hippocampal coherence: Diazepam *reduced* this coherence in all frequency bands and Gamma coherence in the opposing direction, while ketamine significantly *increased* coherence strength for these directions and frequencies. This implies a weakening of prefrontal – hippocampal network coupling under diazepam and strengthening of the same under ketamine. Reductions in intra-hemispheric Theta and Alpha coherence have been observed in awake humans in response to diazepam^40^, while decreased inter-parietal coherence has been reported in rats^41^. The descent into NREM sleep is associated with a reduction in cortical connectivity and eventually a functional decoupling of the prefrontal cortex^42–44^. Our findings therefore indicate that the enhanced induction and maintenance of NREM sleep under diazepam may result from facilitated decoupling of the prefrontal cortex. Conversely, the increased NREM onset latency observed under ketamine may result from a stimulating effect of enhanced prefrontal coupling. These results are particularly exciting as they demonstrate altered communication between *specific* brain structures in response to a *systemically* administered drug. Furthermore, the opposing action of these drugs on prefrontal-hippocampal network coupling as predicted by rPDC are completely in accordance with their opposing actions at the behavioural level.

### Time-resolved qEEG tracks networks during spontaneous alternation

A key challenge in cognitive neuroscience is the assessment of transient cognitive processes and behavioural states. EEG offers high temporal resolution but limitations imposed by analytic methods have squandered this advantage. Here, we demonstrate that time-stationary rPDC analyses during spontaneous alternation in the Y-Maze yielded no overt effect of alternation type on inter-regional connectivity and only a subtle elevation of hippocampal Gamma power. Time-resolved rPDC however demonstrated an astounding level of dynamism in network connectivity within a short time frame. Furthermore, we discovered that correct choices are uniquely accompanied by a specific temporal pattern of Theta coherence on approach to the maze intersection.

The so far undetected pattern of a prefrontal-hippocampal initiation signal expands on the previously suggested role of Theta communication between the two regions, thought to be critical for cognitive function^45^. Synchronization during decision making in working memory tasks^46–48^ was assumed to be primarily hippocampally-driven as suggested by phase locking analysis^45^. However, this assumption is not definitive, particularly when there is an indirect anatomical route between recording sites. Enhanced hippocampal-prefrontal Theta coherence was previously proposed in a reward-based Y-Maze paradigm and a role in learning suggested^49^. Our data now confirms Theta coherence in the same maze location in the absence of a reward, and stipulates that it uniquely precedes ‘correct’ spontaneous alternations, in line with previous observations^48^. Interestingly, a recent study has indicated modulation of hippocampal encoding during route planning via the prefrontal-thalamic-hippocampal pathway^50^. Our data suggest that - regardless of whether a decision is made based on anticipation of reward^49^ or spontaneously - similar neural mechanisms are responsible for executing this choice.

Overall, we propose that our analyses provide compelling evidence that rPDC provides exciting new opportunities for a wide range of neuroscience applications. For example, it is now possible to determine the time course and anatomical conduits through which representations of space encoded by hippocampal place and grid cells^51^ interact with prefrontal executive control functions. Within the realm of neurodegenerative research, hippocampal-prefrontal directional connectivity can also be probed in e.g. AD models and MCI patients. This will ultimately provide valuable information regarding the impact of disease processes on network properties, and may offer a wealth of new biomarkers for diagnosis.

## Electronic supplementary material

Supplementary figures
