# Supplementary material for: Detection of time-, frequency- and direction-resolved communication within brain networks
Source: Sci Rep. 2018 Jan 29;8:1825. doi: 10.1038/s41598-018-19707-1 (PMC5788985; doi:10.1038/s41598-018-19707-1)
Supplement: Supplementary file 1 — Supplementary figures [file 41598_2018_19707_MOESM1_ESM.pdf]

Detection of time-, frequency- and direction-resolved communication within brain networks

(Supplementary figures)

Barry Crouch[a], Linda Sommerlade[b,c], Peter Veselcic[a,e], Gernot Riedel[a],

Björn Schelter[b,c,d], Bettina Platt[a,*]

[a] Institute of Medical Sciences, School of Medicine, Medical Sciences & Nutrition, University of Aberdeen, Foresterhill, Aberdeen AB25 2ZD, United Kingdom

[b] Institute for Complex Systems and Mathematical Biology, University of Aberdeen, King's College, Old Aberdeen AB24 3UE, United Kingdom

[c] Institute for Pure and Applied Mathematics, University of Aberdeen, King's College, Old Aberdeen AB24 3UE, United Kingdom

[d] TauRx Therapeutics Ltd, King Street, Aberdeen, United Kingdom

[e] Present address: AbbVie Deutschland GmbH & Co. KG; Knollstr., 67061 Ludwigshafen, Germany

* Corresponding author. Contact:
Tel: .(+44) 1224 437402
Email: b.platt@abdn.ac.uk

Keywords:

EEG, spectral analysis, connectivity, network, Granger causality, autoregressive modelling, ageing, ketamine, diazepam, Y-maze.

a

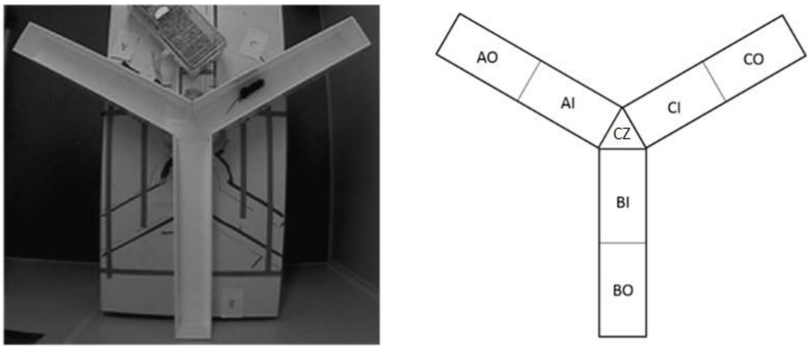

b

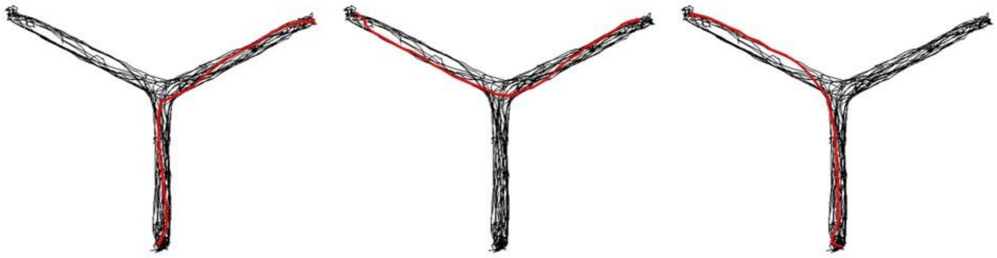

c

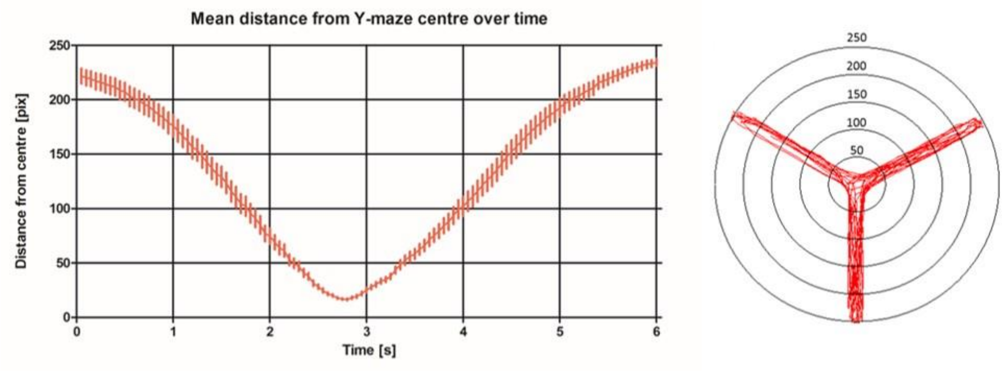

Supplementary Figure 1: Y-Maze apparatus and tracking of mice for EEG analysis.

A: Photograph captured from the overhead camera during a Y-Maze trial: A PLB_1 WT mouse is heading from inner towards outer zone of arm C. The diagram on right provides a map of arm designations as well as inner and outer sub-divisions. Arm A inner zone = AI, Arm A outer zone = AO, etc. The central zone (CZ) is formed by the meeting of the 3 arms. **B:** Red lines indicate exemplary motion path during 6 second periods from which EEG data were extracted, superimposed on track plots (black) of the animal's movement over the full 10 minute trial. Selection of EEG segments based on movement trajectory ensures that behavior of animals is similar in space and time between EEG segments analyzed. **C:** Distance of animals from the centre of the Y-Maze during the 6 seconds of sampled EEG (30 samples in total, mean ± SEM). Distance is measured in pixels [pix] as this is the unit of the motion tracking co-ordinate system. On the right, the maze area and motion paths of all 30 samples are plotted superimposed upon one another (red). A concentric circular grid (black) spaced at 50 pix provides a visual reference for the plot on the left.

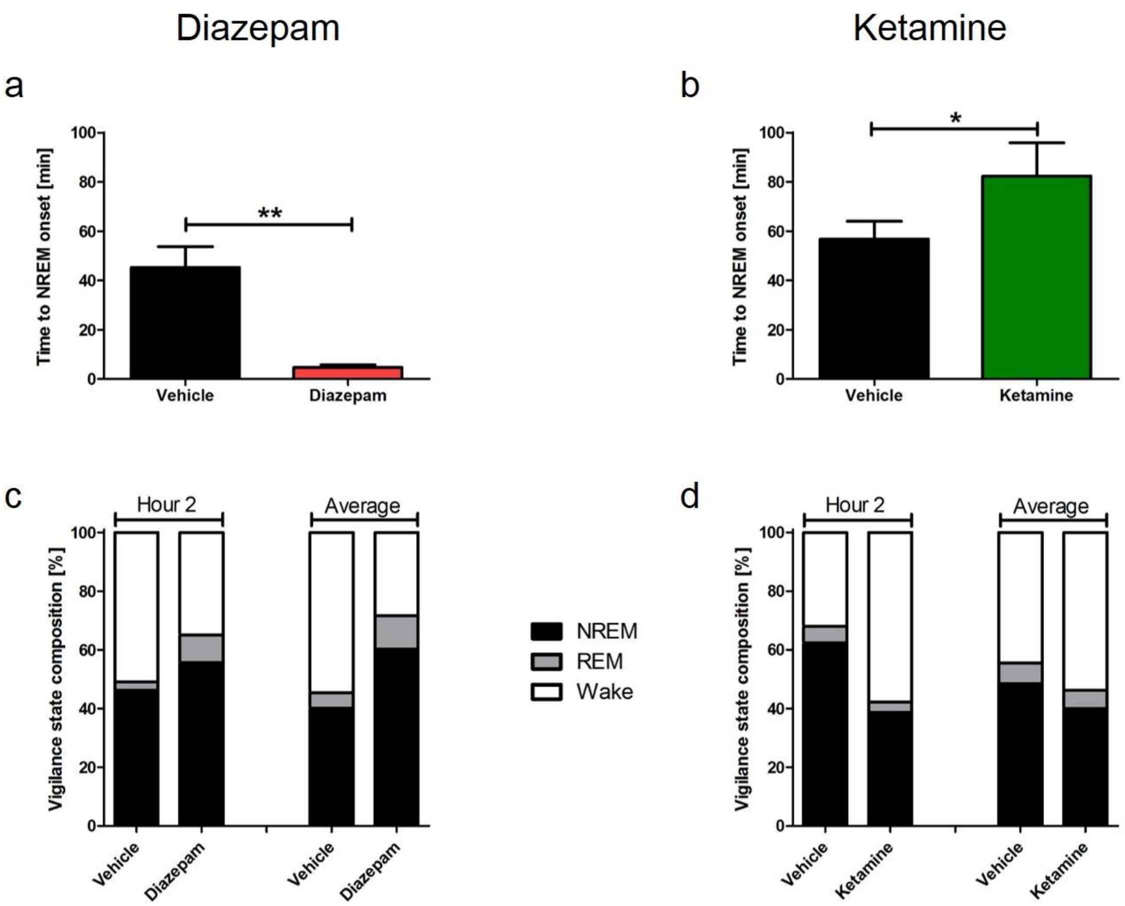

Supplementary Figure 2: Effects of Diazepam and Ketamine on sleep onset.

Plots A and B display the time (mean + SEM time in minutes) between injection and onset of first NREM sleep for mice treated with Diazepam (A - red) and Ketamine (B - green) relative to mice treated with corresponding vehicles (black, within subject design). Asterisks summarize results of paired comparison between drug and vehicle treatment (* = $p<0.05$, ** = $p<0.01$) are provided. Plots C and D represent the vigilance composition [%] for wakefulness (wake), NREM and REM sleep for the second hour and averaged over 0-6 hours following injection of Diazepam / vehicle (C) or Ketamine / vehicle (D).

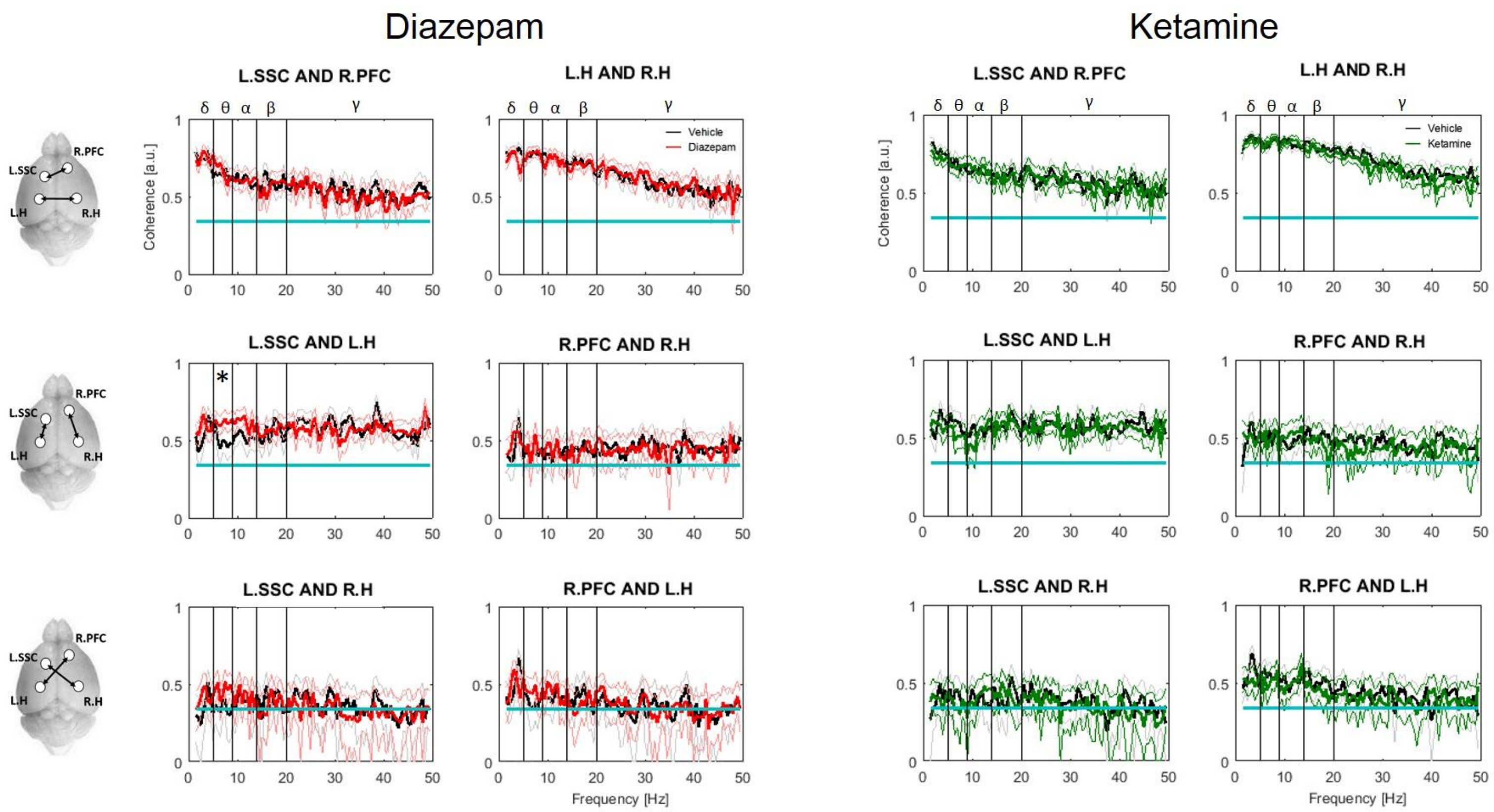

Supplementary Figure 3: Classical coherence analysis of drug effects on connectivity in NREM sleep.

Mean classical coherence strength in arbitrary units [a.u.] observed between all possible channel pairs over the 1-50 Hz range for mice treated with Diazepam (red) or Ketamine (green) relative to respective vehicles (black). Pictograms to left of plots indicate channel pairs are analysed in each row. Subplot titles indicate channels and direction of coherence; R.PFC = right prefrontal, R.H = right parietal (hippocampus), L.H = left parietal (hippocampus) L.SSC = left somatosensory. Blue lines = 95% confidence limit.

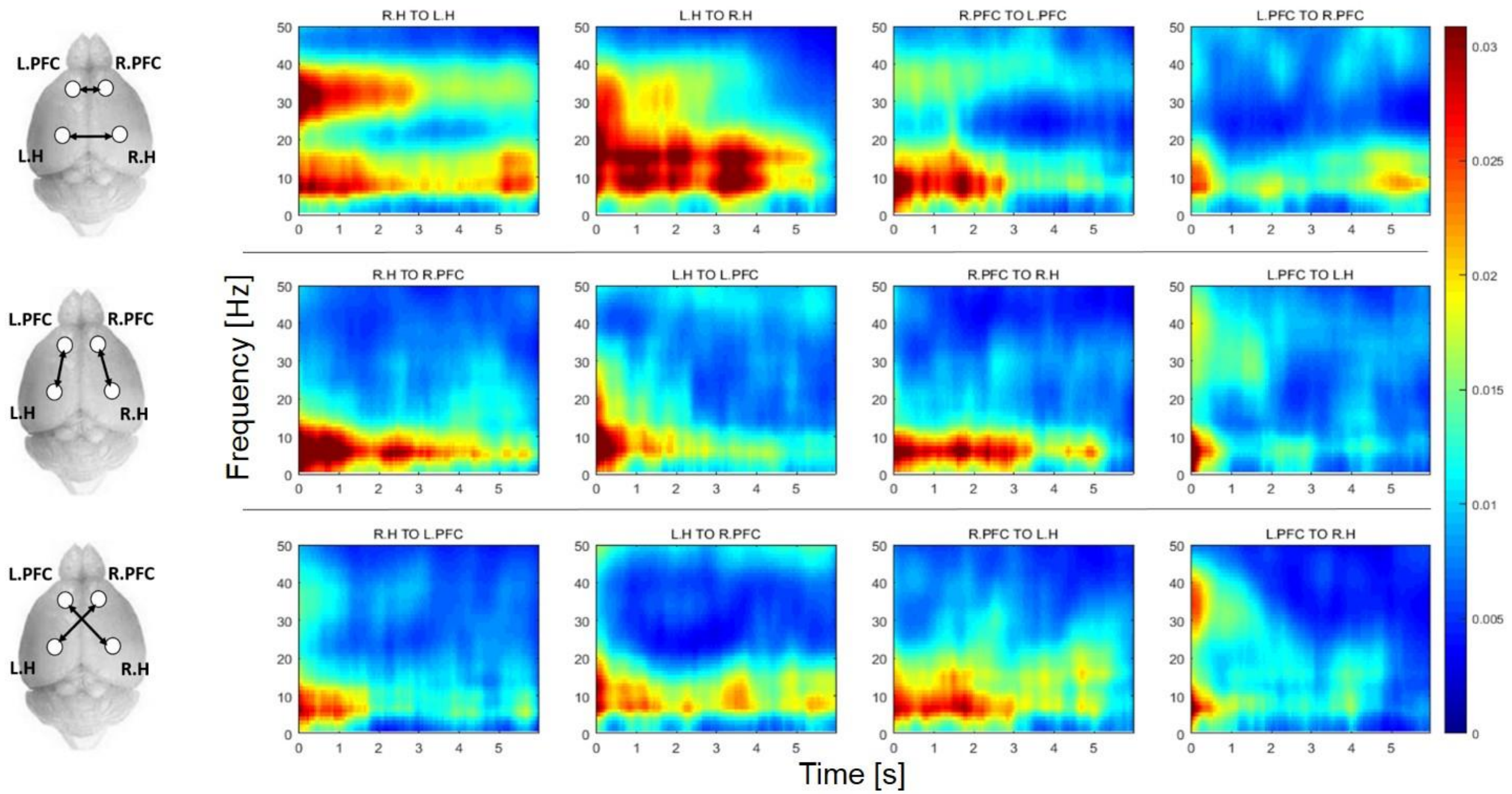

<u>**Supplementary Figure 4: Time resolved connectivity (rPDC) during correct alternations in the Y-Maze.**</u>

Strength of coherence (rPDC) during correct Y-Maze alternations resolved in both time (seconds - X-axis) and frequency (Hz - Y-axis). Colour indicates rPDC strength at each time/frequency point. Colour bar (right) translates rPDC arbitrary units to colour. Pictograms (left) indicate channel pairs analysed in each row (from top to bottom: Interhemispheric Homotypic, ipsilateral heterotypic, interhemispheric heterotypic). Subplot titles indicate channel pair and direction of coherence; left parietal (hippocampus) = L.H, right parietal (hippocampus) = R.H, left prefrontal cortex = L.PFC and right prefrontal cortex = R.PFC. 0-3 seconds corresponds to inward journey from distal end of origin arm towards central zone. 3-6 seconds correspond to outward journey toward distal end of destination arm (see Suppl. Figure 1).

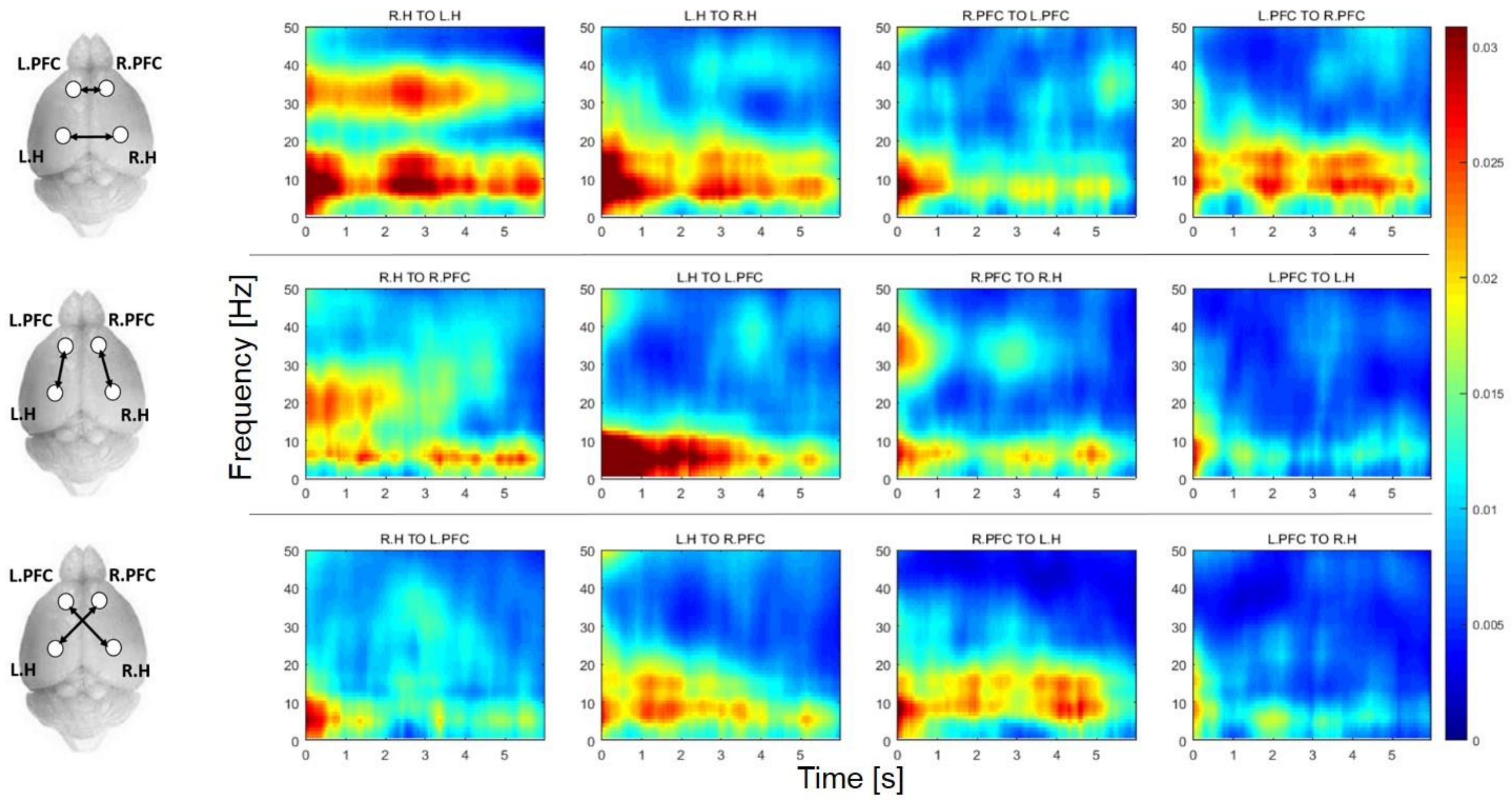

Supplementary Figure 5: Time resolved connectivity (rPDC) during incorrect alternations in the Y-Maze.

Strength of coherence (rPDC) during incorrect Y-Maze alternations resolved in both time (seconds - X-axis) and frequency (Hz - Y-axis). Colour indicates rPDC strength at each time/frequency point. Colour bar (right) translates rPDC arbitrary units to colour. Pictograms (left) indicate channel pairs analysed in each row. Subplot titles indicate channel pair and direction of coherence; left parietal (hippocampus) = L.H, right parietal (hippocampus) = R.H, left prefrontal cortex = L.PFC and right prefrontal cortex = R.PFC.
